# Comparative Pharmacokinetics of three major bioactive components in rats after oral administration of Typhae Pollen-Trogopterus Feces drug pair before and after compatibility

**DOI:** 10.1186/s40199-016-0140-2

**Published:** 2016-01-20

**Authors:** Huiting Zeng, Ping Xue, Shulan Su, Xiaochen Huang, Erxin Shang, Jianming Guo, Dawei Qian, Yuping Tang, Jin-ao Duan

**Affiliations:** Jiangsu Key Laboratory for High Technology Research of TCM Formulae, Nanjing University of Chinese Medicine, Nanjing, 210023 China; Jiangsu Collaborative Innovation Center of Chinese Medicinal Resources Industrialization, Nanjing University of Chinese Medicine, Nanjing, 210023 China; National and Local Collaborative Engineering Center of Chinese Medicinal Resources Industrialization and Formulae Innovative Medicine, Nanjing University of Chinese Medicine, Nanjing, 210023 China; Jiangsu Key Laboratory for TCM Formulae Research, Nanjing University of Chinese Medicine, Nanjing, 210046 People’s Republic of China

**Keywords:** Typhae Pollen-Trogopterus Feces drug pair, Typhaneoside, Vanillic acid, *p*-coumaric acid, Pharmacokinetic, UPLC-TQ/MS

## Abstract

**Background:**

Typhae Pollen (TP) and Trogopterus Feces (TF) are well-known traditional medicine in china which widely used for thousands of years as drug pair called Shixiao San for treatment of blood stasis syndrome, specially shown great efficacy in gynecological disease. Typhaneoside, vanillic acid and *p*-coumaric acid are the main bioactive components of Typhae Pollen. This study was carried out for comparing the pharmacokinetic profile of these three major bioactive components in rats after oral administration of Typhae Pollen-Trogopterus Feces (TP-TF) drug pair before and after compatibility.

**Methods:**

A sensitive and rapid UPLC-TQ/MS method has been developed for simultaneous quantification of the three main bioactive compounds in blood at different time points after oral administration of Typhae Pollen (TP) and the combination with Trogopterus Feces (TF).

**Results:**

There were significant differences of C_max_, T_max_, T_1/2_ and AUC_0~t_ for three bioactive compounds among the groups, for typhaneoside with the most highest plasma concentration of 370.86 ± 315.71 ng/mL and more longer T_max_ in TP-TF co-decoction group (C_M_); for vanillic acid, TP-TF co-decoction group (C_M_) had a good absorption with C_max_ (3870.99 ± 2527.99 ng/mL) and T_max_ (1.47 ± 3.20 h); for *p*-coumaric acid, it had similar pharmacokinetic characteristics with vanillic acid.

**Conclusions:**

The three bioactive components in Typhae Pollen (TP) were simultaneously determined by UPLC-TQ/MS and had a good absorption in rat plasma after the combination with Trogopterus Feces (TF).

**Electronic supplementary material:**

The online version of this article (doi:10.1186/s40199-016-0140-2) contains supplementary material, which is available to authorized users.

## Background

Blood stasis (BS) is considered as a familiar type of clinical symptoms and signs in Traditional Chinese Medicine (TCM) for thousands of years, which is the underlying pathology of many disease processes according to TCM theory. Generally speaking, Blood stasis (BS) refers to retarded blood flow and it is often associated with disruption of heart Qi (vital energy), thus giving rise to a series of hematological disorders such as congestion, hemorrhage, thrombosis, local ischemia (microclots) and even tissue changes [1, 2]. It mainly manifested as pain, lassitude, bleeding, chills and fever, bruise, muscle tension, and some dark blue signs like black rim of eyes [3], women’s blood stasis mostly for gynecology diseases for instance of dysmenorrhea, menoxenia, uterine fibroids in clinic [4].

The drug pair of Typhae Pollen and Trogopterus Feces which we named it for Shixiao San originally came from the *Classified Materia Medica* in volume twenty-two *jinxiao fang*, which was written by Shen-wei Tang in Song dynasty of ancient china [5]. As described in early publications, Typhae Pollen-Trogopterus Feces drug pair is famous for its remarkable and reliable therapeutic actions in a multitude diseases caused by blood stasis such as hyperlipidemia, atherosclerosis, thrombosis, stroke, angina pectoris and gynecological diseases by means of promoting blood circulation and removing stasis [6–8]. Typhae Pollen, known as Puhuang in Chinese is the dry pollen of typhaceae plant *Typha angustifolia* L*.*, *Typha orientalis* Presl., and all species of the genus *Typha* [9]. Recent pharmacological study indicated that Typhae Pollen is proved to possess quite a few of biological activities including inducing uterine contractions, antioxidant, anti-inflammation, wound healing, and etc. [10–12], which owes to the main active ingredients among them like flavonoids, steroids, fatty acids, etc. [13]. Typhaneoside, vanillic acid and *p*-coumaric acid are just the three major bioactive components consist in Typhae Pollen due to their high content and significant bioactivities. Trogopterus feces also called Wulingzhi originally recorded in the *Kaibao Bencao*, and it is the dry feces of *Trogopterus xanthipes* Milne-Edwards (Petauristidae) [14]. It has been reported that the main chemical constituents including terpenoids [15], phenolic acids, sterols, aliphatics, fatty acids, and flavonoids are commonly used in the inhibition of tumor formation, inducing tumor cell apoptosis, antioxidant, reducing antithrombin levels, cytotoxic activity, immunity enhancement, anti-inflammatory activities, etc. [16, 17].

Drug pair, as is less known at abroad, it is the unique combination of two relatively fixed drugs based on theory of TCM in clinic, which nowadays have played a key role increasingly in the development of TCM and captured researcher’s attention for the most fundamental and the simplest form of Chinese drug formulae [18, 19]. As the basic composition units of Chinese drug compatibility, reducing the toxicity and increasing the efficacy of drugs are supposed to be the basis of its efficiency [20]. With an increasing number of researches on drug pairs, works about the possible modes of actions for some famous pairs have obtained a certain achievement, just like Danggui Buxue Decoction (Astragali Radix and Angelicae sinensis Radix) [21] and Taoren-Honghua (Persicae Semen and Carthami Flos) herb pair [22]. More recent study suggested that how the synergistic effects of drug pairs come into being not only by changing the constitution or content of bioactive compounds but also regulating its absorption, distribution, metabolism and excretion (ADME) [23, 24].

According to the previous report, there are numerous studies about Typhae Pollen-Trogopterus Feces drug pair in each aspect while hardly any about the compatibility mechanism of it. Here, based on our early investigations [25, 26], the ultra-performance liquid chromatography coupled with a triple quadrupole electrospray tandem mass spectrometry (UPLC-TQ/MS) method has been performed to compare the pharmacokinetic profile of three major bioactive components including typhaneoside, vanillic acid and *p*-coumaric acid among Typhae Pollen-Trogopterus Feces drug pair before and after compatibility. Achievements of this study were desired to provide beneficial scientific information for revealing the reasonable compatibility of this drug pair and better understanding about its *in vivo* behavior mechanism.

## Methods

### Chemicals, reagents and materials

**Fig. 1:**
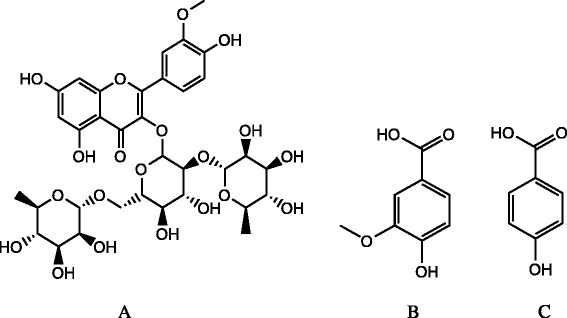
Chemical structures of the reference substances (A. typhaneoside; B. vanillic acid; C. *p*-coumaric acid)

The reference standards of typhaneoside (111573–200603), vanillic acid (110776–200602), *p*-coumaric acid (D-032-120603) and diphenhydramine hydrochloride as internal standards (IS, 130356–200503) were purchased from the National Institute for the Control of Pharmaceutical and Biological Products (Beijing, China), the chemical structures of them are showed in Fig. 1.

Acetonitrile and methanol were of HPLC grade and obtained from Jiangsu Hanbon Science and Technology Co., Ltd. (China) and Tedia (Fairfield, USA), respectively. Formic acid was analytical grade from Merck (Darmstadt, Germany). Ultra-pure water was purified by an EPED super purification system (China). All other reagents were of analytical grade.

Typhae Pollen and Trogopterus Feces were purchased from Nanjing Chinese and Western Pharmaceutical Co., Ltd., and authenticated by the corresponding author. They were within the qualitative and quantitative stipulation of Chinese Pharmacopoeia. The voucher specimens (No.NJUTCM-20090118 for Typhae Pollen and No.NJUTCM-20090119 for Trogopterus Feces) were deposited at the herbarium in Nanjing University of Chinese Medicine, China.

### Apparatus and UPLC-TQ/MS conditions

ACQUITY^TM^ UPLC system, XevoTM TQ mass spectrometry system (Waters Corp., Milford, MA, USA); EPED ultrapure water machine (Nanjing, China); Sartorius BT1250 electronic balance (Sartorius Scientific Instruments Corporation, Beijing, China); CENTRIVAP centrifuge enrichment apparatus (Labconco); ML303 electronic balance (Mettler Toledo Instruments Co., Ltd. Shanghai, China); TDL-80-2B centrifuge (Beckman Coulter, Inc.).

An ACQUITY UPLC BEH C_18_ column (2.1 mm × 100 mm, 1.7 *μ*m, Waters Corp., Milford, MA, USA) was applied and the column temperature was maintained at 35 °C. The mobile phase was composed of A (0.1 % aqueous formic acid) and B (acetonitrile) using a gradient elution of 5–10 % B at 0–1 min, 10–30 % B at 1–6 min, 30–40 % B at 6–7 min, 40–95 % B at 7–8 min, 95–5 % B at 8–9 min, 95 % B at 9–10 min with a flow rate set at 0.4 mL/min. The auto-sampler was conditioned at 4 °C and the injection volume was 5 *μ*L.

Mass spectrometry detection was performed by using a Xevo Triple Quadrupole MS (Waters Corp., Milford, MA) equipped with an electrospray ionization source (ESI). The ESI source was set in positive ionization mode. The scanning mode was set multiple reaction monitoring (MRM) mode. Parameters set in the source were as follows: the capillary voltage at 1 kV; sampling cone voltage of 30 V; source temperature 15 °C; desolvation temperature 550 °C; dwell time was automatically set by MassLynx (Waters Corp., Milford, MA, USA). The cone voltage and collision energy optimized for each analyte and selected values are given in Table 1.

**Table 1:** The optimum mass spectrometry conditions for three compounds and IS

Analytes	Ionizationmode	MRM transitions (precursor-product)	Cone voltage (V)	Collision energy (eV)
Typhaneoside	ES^+^	771.0319 → 317.0719	18	26
Vanillic Acid	ES^+^	168.8404 → 65.1300	16	20
*P*-coumaric Acid	ES^+^	164.8404 → 91.0910	16	26
Diphenhydramine Hydrochloride	ES^+^	256.2127 → 167.1291	12	16

### Preparation of calibration standards and quality control (QC) samples

The mixture of standard stock solution containing above three compounds were prepared in methanol and giving a final concentrations of 28.2 *μ*g/mL for typhaneoside, 25.7 *μ*g/mL for vanillic acid, and 26.5 *μ*g/mL for *p*-coumaric acid, respectively. The mixture stock solution was serially diluted with methanol to provide working standard solutions of desired concentration of 0.282 mg/mL for typhaneoside, 0.257 mg/mL for vanillic acid, and 0.265 mg/mL for *p*-coumaric acid, respectively. The IS stock solution were also prepared in methanol for diphenhydramine hydrochloride (IS) of 1.04 *μ*g/mL.

Calibration standards and quality control (QC) samples were prepared as following: the mixture of standard working solution was diluted into eight different concentration gradients, and given the final concentration of 0.142 ~ 7100 ng/mL for typhaneoside, 0.104 ~ 5200 ng/mL for vanillic acid, 0.102 ~ 5100 ng/mL for p-coumaric acid. Quality control (QC) samples at low, middle and high concentrations were 3.55, 71.0, 710 ng/mL for typhaneoside, 2.60, 52.0, 260 ng/mL for vanillic acid and 2.55, 51.0, 255 ng/mL for p-coumaric acid. All solutions were stored at 4 °C and brought to room temperature before use. QC samples were stored at −20 °C before analysis.

### Preparation of drug extraction and sample solutions

The raw materials of Typhae Pollen (200 g) for single extract and Typhae Pollen–Trogopterus Feces (200 g + 200 g) for co-decoction (two drugs decoct together) and mixed decoction (two drugs decoct separately and then mix) was accurately weighed, extracted with boiling water (1:10) for 2 h, and then extracted with boiling water (1:8) for 2 h. The filtrates were combined and solvent was removed under reduced pressure in a rotary evaporator to reach a certain volume at the ratio of 1:1 (w/w, weight of all constituting drugs and the extract filtrates) for the TP single drug extraction; 2:1 for the TP-TF co-decoction and TP-TF mixed decoction. The contents of three compounds measured quantitatively by UPLC were 167.73, 168.37, 153.32 *μ*g/mL for typhaneoside; 51.83, 48.67, 45.02 *μ*g/mL for vanillic acid and 36.62, 35.67, 33.12 *μ*g/mL for *p*-coumaric acid in single extract, co-decoction and mixed decoction, respectively.

A 200 *μ*L aliquot of plasma sample was added with 10 *μ*L diphenhydramine hydrochloride working solution and 600 *μ*L of methanol. After vortex for 2 min and centrifugation at 13,000 rpm for 10 min, the supernatant was transferred to another 1.5 mL tube and concentrated in the centrifuge enrichment apparatus at 37 °C.

Finally, each residue was reconstituted in 200 *μ*L 70 % methanol, then vortexed for 3 min and centrifuged at 13,000 rpm for 10 min. 5 *μ*L of supernatant was injected into the UPLC-TQ/MS system for analysis.

### Validation of the HPLC method

#### Specificity, linearity and LLOQ

The specificity of the method was evaluated by comparing chromatograms of blank plasma sample, blank plasma sample spiked with reference standards and internal standards, and plasma sample after oral administration of Typhae Pollen-Trogopterus Feces co-decoction for 30 min.

The linearity of each calibration curve was determined by plotting the peak area ratio (Y) of analytes to corresponding IS versus the nominal concentration (X) of analytes with weighted (1/*X*^2^) least square linear regression. The lower limit of quantification (LLOQ) was determined as the lowest concentration with a signal-to-noise (S/N) ratio of 10.

#### Precision and accuracy

Accuracy and intra- and inter-day precision of the established method were evaluated by QC samples at low, medium and high concentrations (six samples for each) on 3 consecutive validation days. The precision expressed by relative standard deviation (RSD%), and the accuracy by relative percentage error (%).

#### Extraction recovery, matrix effect and stability

The extraction recoveries of analytes were determined by comparing the peak responses of three QC samples (six samples for each) in the post-extraction spiked samples to that acquired from pre-extraction spiked samples at equivalent concentrations. The matrix effect was evaluated by comparing the peak responses of samples where the extracted matrix was spiked with standard solutions to those obtained from neat standard solutions at equivalent concentrations.

The stability of the analytes in rat plasma was assessed by analyzing QC samples at three concentration levels (six samples for each) under different condition. Three QC samples were tested for pre-treatment, post-treatment and three freeze-thaw cycles at room temperature for 12 h, refrigerated (4 °C) for 24 h and repeatedly frozen and thawed for three times at −80 °C, respectively.

#### Pharmacokinetic studies

All experiments were performed on female Sprague–Dawley (SD) rats, weighing 220–250 g, obtained from Shanghai Slac Laboratory Animal Co., Ltd. (Shanghai, China). They were kept in plastic cages at 22 ± 2 °C and a relative humidity of 50–65 %, with free access to pellet food and water on a 12 h light/dark cycle. Animal welfare and experimental procedures strictly conformed to the Guide for the Care and Use of Laboratory Animals [27] and the related ethics regulations of Nanjing University of Chinese Medicine.

For pharmacokinetic studies, the SD rats were divided into four groups randomly (*n* = 6 per group), and housed with unlimited access to food before the experiment while water available. Each group except the control group was oral administration of TP (C_N_) single drug decoction, TP-TF co-decoction (C_M_) and TP-TF mixed decoction (C_B_) at a dosage of 5 g/kg, 10 g/kg, 10 g/kg body weight, respectively. Blood samples (0.5 mL) were collected at certain time points and placed at a 1.5 mL tube before oral (0 min) and after oral (5, 15, 30, 45, 60, 120, 240, 360, 480, 720 and 1440 min) administration. Afterwards all the blood samples were centrifuged at 13,000 rpm for 10 min and stored at −80 °C until analysis.

## Results and discussion

### Validation of the quantitative analysis

**Fig. 2:**
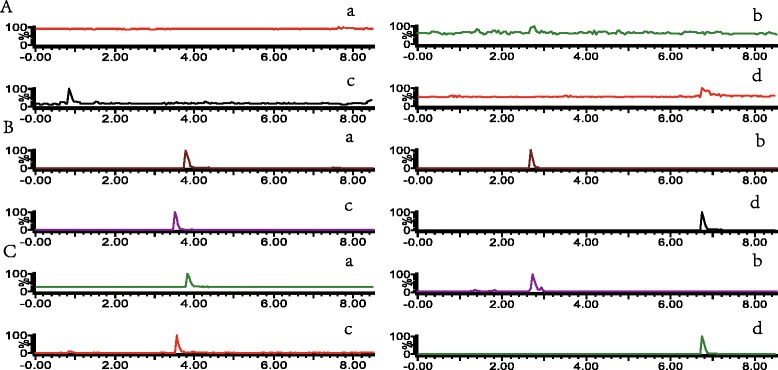
Representative MRM chromatograms of the three components in rats: A. blank plasma. B. blank plasma samples spiked with reference standards and internal standards. C. plasma sample after oral administration of Typhae Pollen- Trogopterus Feces co-decoction for 30 min. Note: a. typhaneoside; b. vanillic acid;c. *p*-coumaric acid; d. diphenhydramine hydrochloride

Fig. 2 shows the typical MRM chromatograms of blank plasma sample, blank plasma sample spiked with reference standards and internal standards, and plasma sample after oral administration of Typhae Pollen-Trogopterus Feces co-decoction for 30 min. No interference peaks were observed at the retention times of analytes and IS in any plasma that used for analysis, the method presented good specificity.

The linear regression equation, correlation coefficient and LLOQ for typhaneoside, vanillic acid and p-coumaric acid in rat plasma samples are shown in Additional file 1. The calibration curves exhibited good linearity with correlation coefficients (r) and the LLOQ were sufficient for pharmacokinetic studies of these analytes.

Intra-day, inter-day accuracy and precision of the three compounds in rat plasma samples are presented in Additional file 2, which showed the method with good accuracy and precision. All the results were found to be within the accepted variable limits.

Extraction recoveries and matrix effect of the three compounds were evaluated by analyzing QC samples at low, medium and high concentrations with six replicates. As the results are shown in Additional file 3, the mean recovery of the analytes was within 70 to 91 % (RSD was less than 11). The corresponding matrix effect ranged from 76 to 106 % (RSD was less than 9).

The stability of the analytes in rat plasma samples was evaluated under different conditions. Deviations for the peak area of the three components were within 15 %, which indicated good stability under the experimental conditions.

### Pharmacokinetic study

**Fig. 3:**
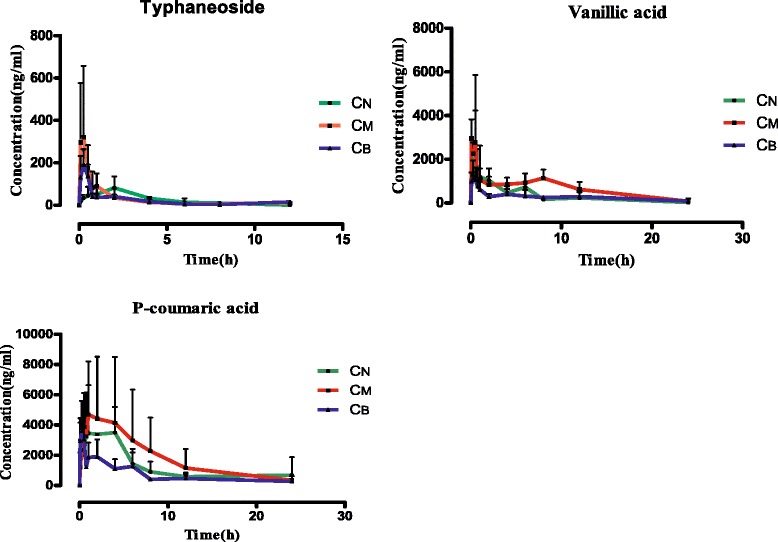
Mean plasma concentration-time curves of three compounds after oral administration with TP (C_N_), TP-TF co-decoction (C_M_) and TP-TF mixed decoction (C_B_) (*n =* 6)

The mean plasma concentration-time profiles of typhaneoside, vanillic acid and *p*-coumaric acid were determined after oral administration with different compatibility of Typhae Pollen-Trogopterus Feces drug pair in rats, the concentration-time curves are presents in Fig. 3, and the noncompartment model pharmacokinetic parameters including maximum plasma concentration (C_max_), time to reach the maximum concentrations (T_max_), half-time (T_1/2_), area under concentration-time curve (AUC _0~t_) are summarized in Table 2.

**Table 2:** Noncompartment model pharmacokinetic parameters of the three compounds after oral administration with extracts (*n* = 6)

Compounds	Pharmacokinetic parameters	TP	TP-TF co-decoction	TP-TF mixed decoction
Typhaneoside	C_max_ (ng/mL)	76.39 ± 53.21	370.86 ± 315.71^*^	214.32 ± 73.72^**^
	T_max_ (h)	2.95 ± 2.88	0.24 ± 0.15^*^	0.21 ± 0.08
	T_1/2_ (h)	3.35 ± 2.58	1.91 ± 1.26	1.37 ± 0.54
	AUC_0~t_ (ng/h/mL)	268.31 ± 167.71	333.46 ± 191.84	275.62 ± 206.06
Vanillic acid	C_max_ (ng/mL)	2211.68 ± 1187.17	3870.99 ± 2527.99	1447.29 ± 500.99
	T_max_ (h)	1.25 ± 0.71	1.47 ± 3.20	0.17 ± 0.09^**^
	T_1/2_ (h)	3.69 ± 0.87	11.25 ± 13.61	8.88 ± 3.76
	AUC_0~t_ (ng/h/mL)	10445.53 ± 4148.68	14137.57 ± 4540.37	6193.78 ± 4499.17
*P*-coumaric acid	C_max_ (ng/mL)	4901.39 ± 1887.30	5110.22 ± 3671.26	4189.45 ± 844.37
	T_max_ (h)	1.08 ± 1.67	1.42 ± 1.39	0.46 ± 0.40
	T_1/2_ (h)	3.67 ± 1.82	10.08 ± 7.86	10.08 ± 10.23
	AUC_0~t_ (ng/h/mL)	36881.01 ± 25783.25	41512.56 ± 41763.28	17689.11 ± 3882.21

^*^ *P* < 0.05,^**^ *P* < 0.01versus TP sole administration

From the datas, it indicated that typhaneoside, vanillic acid and *p*-coumaric acid could be detected immediately by UPLC-TQ/MS, which revealed superior absorption of the three compounds, and significant differences existed among three different compatibilities, all comparing to the control group with TP single drug extraction by *t*-test. For typhaneoside, the peak plasma concentration (C_max_) was 370.86 ± 315.71 ng/mL (*P* < 0.05) in TP-TF co-decoction group (C_M_) and 214.32 ± 73.72 ng/mL (*P* < 0.01) in TP-TF mixed decoction group (C_B_), it was significantly higher than TP single drug extraction (C_N_), though in the previous report it was described as a low bioavailability [28]. What’s more, the time to reach maximum concentrations (T_max_) were 0.24 ± 0.15 h (*P* < 0.05) for TP-TF co-decoction group (C_M_) and 0.21 ± 0.08 h for TP-TF mixed decoction group (C_B_), which implies highly uptake of this compound in rats plasma.

As a kind of common compound in many Chinese medicines, vanillic acid in TP-TF co-decoction group (C_M_) had a good absorption after oral administration for the C_max_ was 3870.99 ± 2527.99 ng/mL and T_max_ was 1.47 ± 3.20 h. In TP-TF mixed decoction group (C_B_) the C_max_ was 1447.29 ± 500.99 ng/mL and T_max_ 0.17 ± 0.09 h (*P* < 0.01). In addition, it is not difficult to find that T_max_ and T_1/2_ in co-decoction group (C_M_) were longer than the two others, and double peak phenomenon was observed at the same time, it probably attribute to the enterohepatic circulation in drug metabolism or the interactivity in Typhae pollen-Trogopterus Feces drug pair. Similarly as vanillic acid, *p*-coumaric acid was also absorbed well, C_max_ was 5110.22 ± 3671.26 ng/mL in TP-TF co-decoction group (C_M_) in addition to its T_max_ was somewhat longer. It may be in virtue of its hydrolyzed diffusion *in vivo* after oral administration with decoction [29], and we can see double peaks clearly on the concentration-time curves, all these hypothesis need further investigations.

When making comparison between TP-TF co-decoction group (C_M_) and TP-TF mixed decoction group (C_B_), C_max_ and AUC_0~t_ of the three active ingredients in TP-TF co-decoction group (C_M_) are higher than TP-TF mixed decoction group (C_B_). It proved that the absorption was increased after compatibility and the duration of drug action was prolonged for T_1/2_ of the three compounds in TP-TF co-decoction group (C_M_) were longer than TP-TF mixed decoction group (C_B_). Generally speaking, the TP-TF co-decoction group (C_M_) showed more advantages of bioavailability for bioactive components.

The results indicated that after Typhae Pollen and Trogopterus Feces used in combination as a drug pair, the three bioactive compounds typhaneoside, vanillic acid and *p*-coumaric acid were well absorbed and slowly eliminated in rats, thus to enhance and prolong clinical efficacy, which may be due to the synergic action between Typhae Pollen and Trogopterus Feces. According to traditional Chinese medicine theory, the compatibility mechanisms of drug pairs are not only arbitrary plus of two drugs but also the regularity of active components *in vivo* [30]. When Typhae Pollen combined with Trogopterus Feces, the dissolution of chemical ingredients were increased [31], followed by the good absorption of three components in TP. We speculated that it’s probably the volatile components in TF which enhanced the absorption of the three components in TP [32, 33]. Furthermore the higher uptake and slower elimination of three compounds in TP by drug pair administration were related to the interaction between drugs mediated by transport proteins, metabolic enzymes, or plasma protein binding, etc. The compatibility mechanism of TP-TF still deserves further research.

## Conclusion

In this paper, the simple, rapid and sensitive UPLC-TQ/MS method was successfully applied to detect three bioactive components in Typhae Pollen before and after the combination with Trogopterus Feces simultaneously. It was the first study to report about the pharmacokinetic parameters of typhaneoside, vanillic acid and *p*-coumaric acid in TP-TF after oral administration. Results indicated that the three compounds have better absorption and slower elimination after Typhae Pollen and Trogopterus Feces combination. The *in vivo* changes of three main active substances was helpful for finding the compatibility principles of TP-TF and clarifying its rational compatibility, thus for better clinical application and research about relative TCM formulas.

## Additional files

Additional file 1:
**The regression equations, correlation coefficient, linear ranges and LLOQ of three analytes (** ***n*** **= 6).** (DOC 21 kb) Additional file 2:
**Intra-day, inter-day accuracy and precision of three analytes (** ***n*** **= 6).** (DOC 21 kb) Additional file 3:
**Recoveries and matrix effects of three analytes (** ***n*** **= 6).** (DOC 21 kb)

## Abbreviations

TP: Typhae Pollen
TF: Trogopterus Feces
C_N_: TP single extract group
C_M_: TP–TF co-decoction group
C_B_: TP–TF mixed decoction group
BS: Blood stasis
TCM: Traditional Chinese Medicine
ADME: Absorption, distribution, metabolism and excretion
UPLC-TQ/MS: Ultra-performance liquid chromatography coupled with a triple quadrupole electrospray tandem mass spectrometry
ESI: Electrospray ionization source
MRM: Multiple reaction monitoring
QC: Quality control
LLOQ: Lower limit of quantification
SD: Sprague–Dawley
