# Supplementary material for: Comparative Pharmacokinetics of three major bioactive components in rats after oral administration of Typhae Pollen-Trogopterus Feces drug pair before and after compatibility
Source: Daru. 2016 Jan 20;24:2. doi: 10.1186/s40199-016-0140-2 (PMC4719211; doi:10.1186/s40199-016-0140-2)
Supplement: Additional file 1: — The regression equations, correlation coefficient, linear ranges and LLOQ of three analytes ( n = 6). (DOC 21 kb) [file 40199_2016_140_MOESM1_ESM.docx]

**Additional file 1**

The regression equations, correlation coefficient, linear ranges and LLOQ of three analytes (n=6)

| Compound | Linear regression equation | r^2^ | Range(ng/mL) | LLOQ(ng/ml) |
| --- | --- | --- | --- | --- |
| Typhaneoside | *Y* = 0.00043 *X* + 0.00323 | 0.996 | 3.55~3550 | 0.71 |
| Vanillic acid | *Y* = 0.00053 *X* + 0.00017 | 0.993 | 0.52~260.0 | 0.52 |
| *P*-coumaric acid | *Y* = 0.00023 *X* + 0.00011 | 0.999 | 0.51~255.0 | 0.51 |
