# Supplementary material for: Comparative Pharmacokinetics of three major bioactive components in rats after oral administration of Typhae Pollen-Trogopterus Feces drug pair before and after compatibility
Source: Daru. 2016 Jan 20;24:2. doi: 10.1186/s40199-016-0140-2 (PMC4719211; doi:10.1186/s40199-016-0140-2)
Supplement: Additional file 2: — Intra-day, inter-day accuracy and precision of three analytes ( n = 6). (DOC 21 kb) [file 40199_2016_140_MOESM2_ESM.docx]

**Additional file 2**

Intra-day, inter-day accuracy and precision of three analytes (n=6)

| Compounds | Concentration (ng/mL) | Intra-day | | Inter-day | |
| --- | --- | --- | --- | --- | --- |
|  |  | Accuracy (%) | Precision (RSD,%) | Accuracy (%) | Precision (RSD,%) |
| Typhaneoside | 710 | 95.98±4.47 | 4.66 | 94.92±5.24 | 5.52 |
|  | 71 | 104.09±7.31 | 7.02 | 98.88±6.15 | 6.22 |
|  | 3.55 | 86.47±6.76 | 7.82 | 103.38±4.78 | 4.63 |
| Vanillic acid | 260 | 104.43±5.35 | 5.13 | 105.05±5.68 | 5.42 |
|  | 52 | 90.78±12.05 | 13.28 | 94.826±9.98 | 10.53 |
|  | 2.6 | 103.46±4.23 | 4.09 | 95.00±8.07 | 8.5 |
| *P*-coumaric acid | 255 | 93.31±7.67 | 8.22 | 94.60±6.79 | 7.18 |
|  | 51 | 92.52±2.92 | 3.16 | 84.72±5.37 | 6.34 |
|  | 2.55 | 106.27±5.88 | 5.54 | 92.94±5.49 | 5.91 |
