# Supplementary material for: Comparative Pharmacokinetics of three major bioactive components in rats after oral administration of Typhae Pollen-Trogopterus Feces drug pair before and after compatibility
Source: Daru. 2016 Jan 20;24:2. doi: 10.1186/s40199-016-0140-2 (PMC4719211; doi:10.1186/s40199-016-0140-2)
Supplement: Additional file 3: — Recoveries and matrix effects of three analytes ( n = 6). (DOC 21 kb) [file 40199_2016_140_MOESM3_ESM.docx]

| Compounds | Concentration (ng/mL) | Extraction  recovery | RSD% | Matrix effect | RSD% |
| --- | --- | --- | --- | --- | --- |
| Typhaneoside | 710 | 83.63± 4.76 | 5.69 | 86.74 ± 6.34 | 7.31 |
|  | 71.0 | 85.41 ± 5.61 | 6.57 | 89.67 ± 5.55 | 6.19 |
|  | 3.55 | 79.77 ± 2.74 | 3.43 | 81.39 ±3.61 | 4.44 |
| Vanillic acid | 260 | 79.76 ± 5.32 | 6.67 | 81.86 ± 3.69 | 4.51 |
|  | 52.0 | 77.66 ± 7.82 | 10.07 | 83.27 ± 6.53 | 7.84 |
|  | 2.6 | 81.30 ±5.71 | 7.02 | 79.63 ±3.82 | 4.8 |
| *P*-coumaric acid | 255 | 82.66 ± 6.77 | 8.19 | 88.66 ± 6.79 | 7.66 |
|  | 51 | 81.46 ± 4.78 | 5.87 | 85.72 ± 4.91 | 5.73 |
|  | 2.55 | 87.39 ±5.63 | 6.44 | 77.43 ±5.77 | 7.45 |

**Additional file 3**

Recoveries and matrix effects of three analytes (n=6)
